# Supplementary material for: Disorganized functional architecture of amygdala subregional networks in obsessive-compulsive disorder
Source: Commun Biol. 2022 Nov 4;5:1184. doi: 10.1038/s42003-022-04115-z (PMC9636402; doi:10.1038/s42003-022-04115-z)
Supplement: Supplementary file 2 — Supplementary Information [file 42003_2022_4115_MOESM2_ESM.pdf]

Supplementary Information

1. Cluster quality metrics

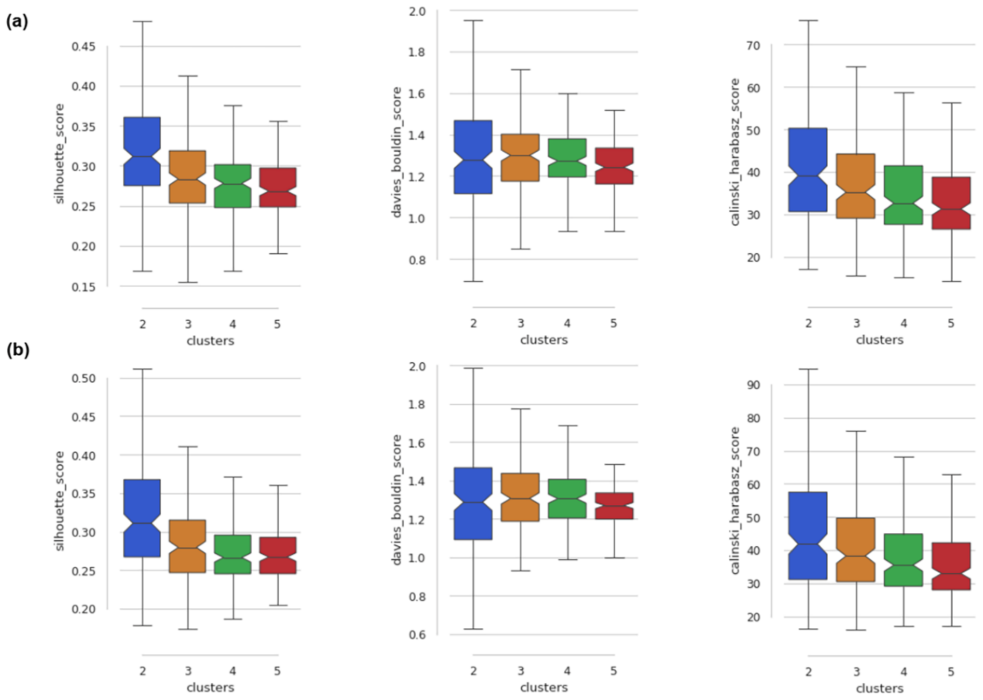

Supplementary Figure 1 Internal validity metrics for all tested solutions (k=2, 3, 4, 5) of the left amygdala (a) and right amygdala (b). The Silhouette index (left) and the Calinski–Harabasz index (right) indicate a better fit through a higher score, whereas the Davies–Bouldin index (middle) is better when lower. The Silhouette and Calinski–Harabasz indices suggested the two-cluster solution to best fit the input data, whereas the Davies–Bouldin index instead suggested the five-cluster solution to fit better. Combined, we chose the two-cluster solution. Center line, median; box limits, quartiles.

2. Spatial correlation with standard template

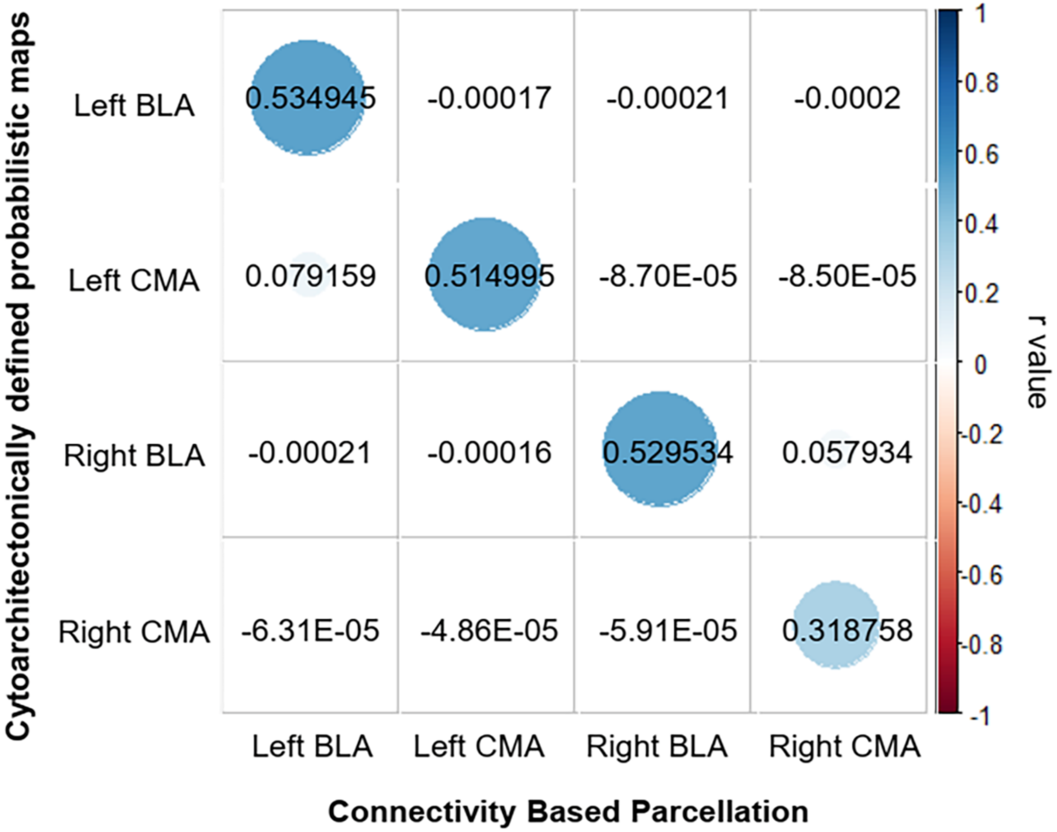

Supplementary Figure 2 Spatial correlation between the BLA/CMA clusters obtained by CBP technique and their respective parcellation from cytoarchitectonically defined probabilistic maps of the amygdala.

3. Similarity between the individual clusterings and the group clustering

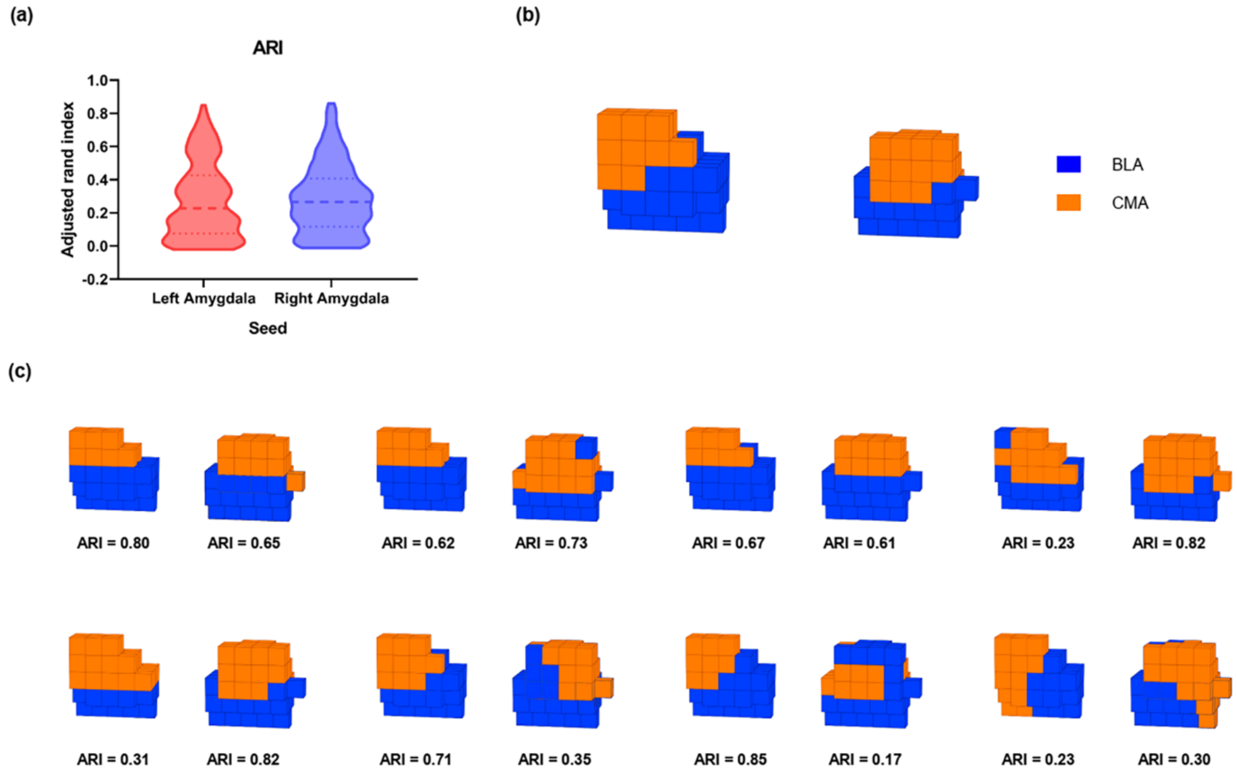

Supplementary Figure 3 (a) Adjusted rand index (ARI) scores between the individual subject clustering results and the group-level clustering result for k = 2. Center line, median; dotted line, quartiles. (b) Group-level clustering result. (c) Several examples of subject-level clustering for the amygdala 2-cluster solution and their adjusted rand index values to the group-level clustering.

4. Main effect of diagnosis on amygdala functional connectivity

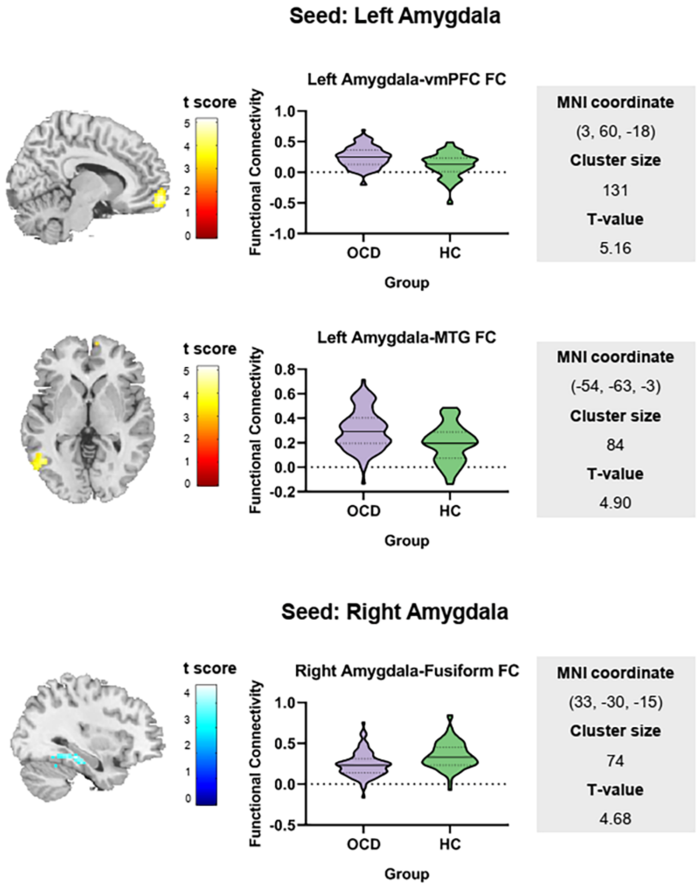

Supplementary Figure 4 Brain regions showing significant group differences in resting-state functional connectivity with the amygdala. A color bar indicating t values is shown. Center line, median; dotted line, quartiles. MTG, middle temporal gyrus; vmPFC, ventromedial prefrontal cortex.

5. Validation of fMRI preprocessing with smooth (6 mm FWHM Gaussian kernel)

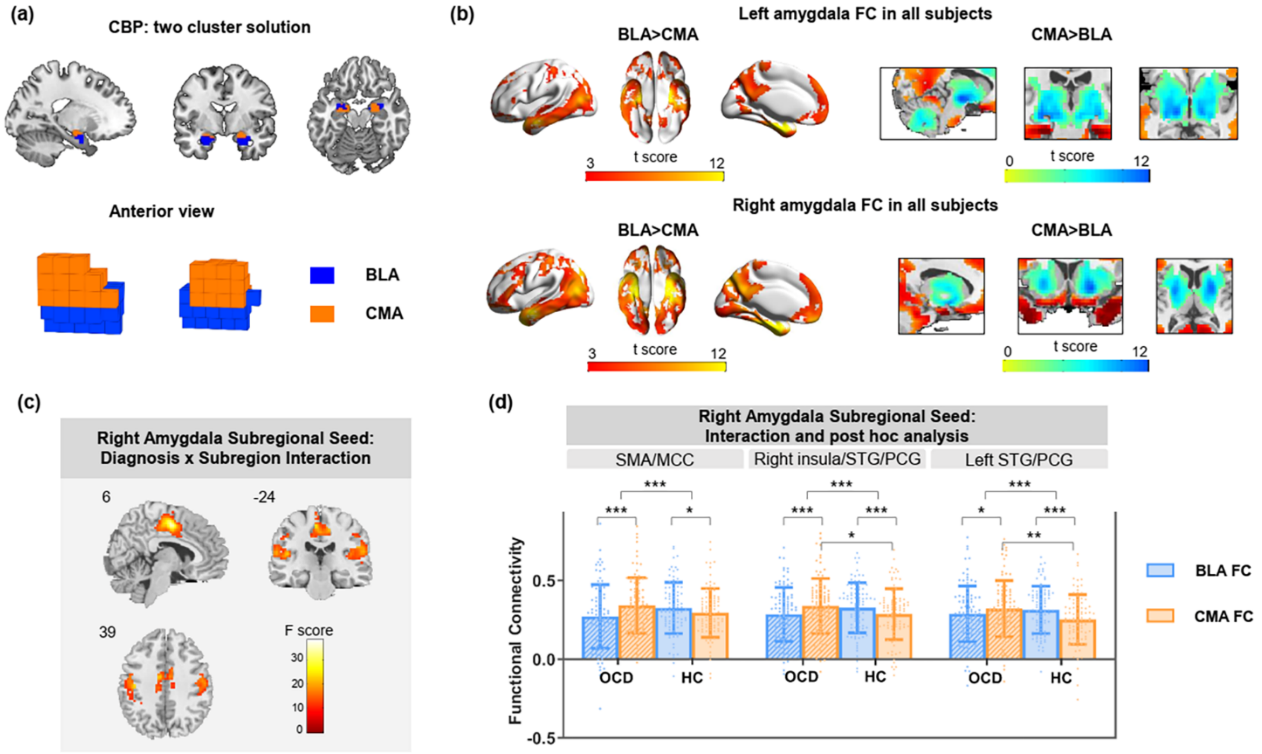

Supplementary Figure 5 (a) The two-cluster solution of CBP. (b) The basolateral cluster (BLA) connectivity was primarily cortical, whereas the centromedial cluster (CMA) connectivity was primarily subcortical. (c) Brain regions that show significant diagnosis × subregion interaction. (d) Interaction effects and post hoc analyses. Error bars represent standard deviation. MCC, midcingulate cortex; PCG, postcentral gyrus; SMA, supplementary motor area; STG, superior temporal gyrus.

6. Validation of fMRI preprocessing without smooth

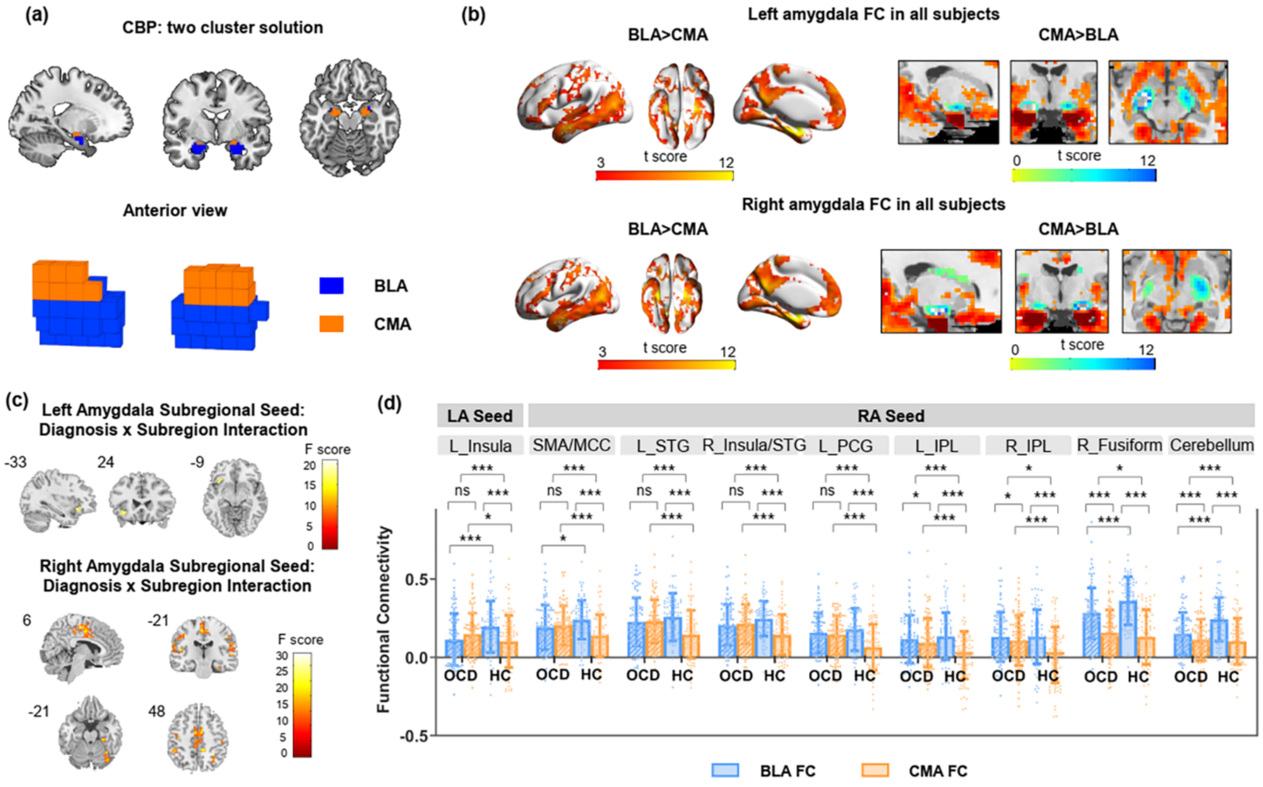

Supplementary Figure 6 (a) The two-cluster solution of CBP. (b) The basolateral cluster (BLA) connectivity was primarily cortical, whereas the centromedial cluster (CMA) connectivity was primarily subcortical. (c) Brain regions that show significant diagnosis × subregion interaction. (d) Interaction effects and post hoc analyses. Error bars represent standard deviation. One OCD and one HC were excluded for left amygdala analysis, and one OCD and three HC were excluded for right amygdala analysis due to software failure. IPL, inferior parietal lobule; LA, left amygdala; RA, right amygdala.

7. Validation of fMRI preprocessing with scrubbing

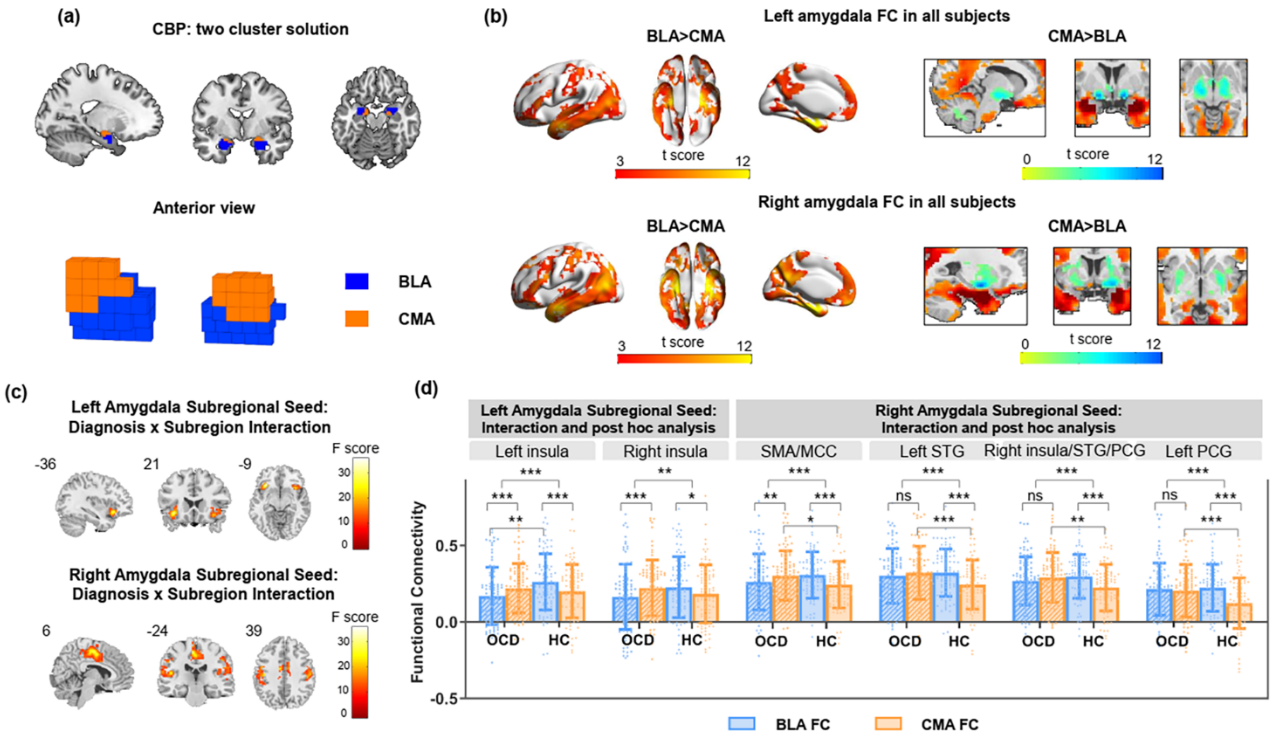

Supplementary Figure 7 (a) The two-cluster solution of CBP. (b) The basolateral cluster (BLA) connectivity was primarily cortical, whereas the centromedial cluster (CMA) connectivity was primarily subcortical. (c) Brain regions that show significant diagnosis × subregion interaction. (d) Interaction effects and post hoc analyses. Error bars represent standard deviation. One OCD with < 4min of scrubbed data was excluded for analyses.

8. Validation of BLA/CMA standard parcellation

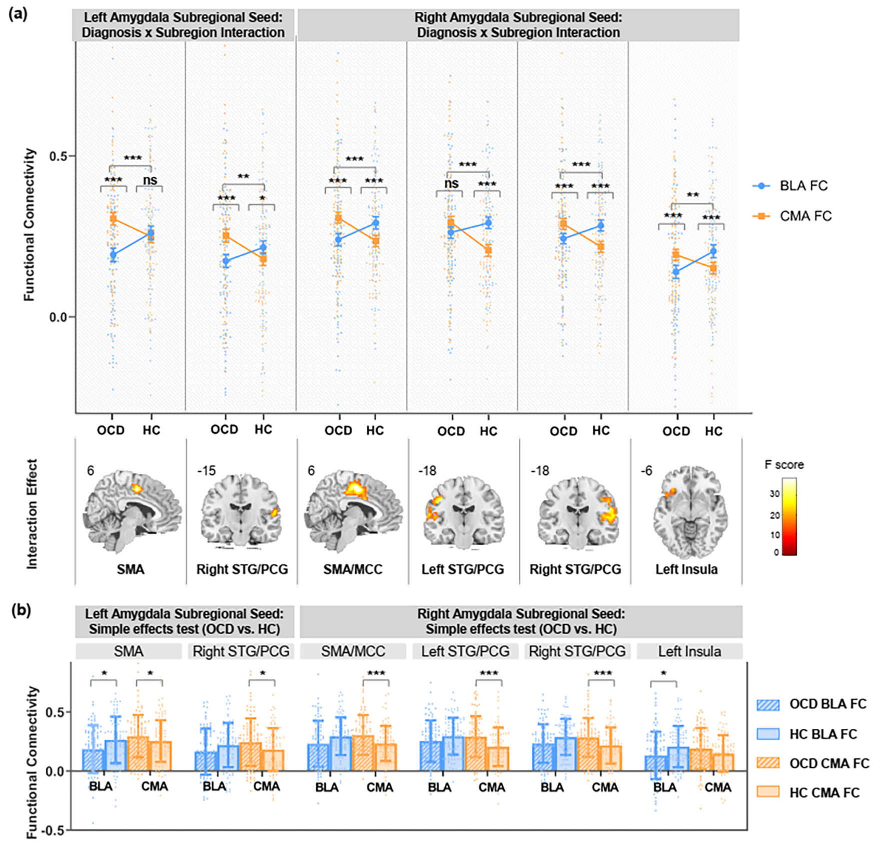

Supplementary Figure 8 (a) Significant interactions between diagnosis (OCD vs. HC) and subregion (BLA vs. CMA) computed separately in left and right amygdala. (b) Simple effects tests comparing functional connectivity (FC) between OCD versus HC in each subregion. Error bars represent standard deviation. Significance is indicated for uncorrected *p < .05; **p < .01; ***, p < .005. MCC, midcingulate cortex; PCG, postcentral gyrus; SMA, supplementary motor area; STG, superior temporal gyrus.

Supplementary Table 1 Brain regions that show significant interactions between diagnosis (OCD vs. HC) and subregion (BLA vs. CMA) computed separately in amygdala from each hemisphere.

Region	Hemisphere	Voxels	F-value	Peak Voxel MNI Coordinates			$p_{FWE\text{-}corrected}$
				X	Y	Z	
**Left Amygdala Seed: diagnosis × subregion interaction**							
Insula	L	90	35.57	-33	24	-9	.005
Insula	R	65	23.43	36	18	-9	.020
**Right Amygdala Seed: diagnosis × subregion interaction**							
Supplementary motor area/Midcingulate cortex	L/R	476	38.44	9	-6	48	<.001
Superior temporal gyrus	L	233	27.04	-45	-24	15	<.001
Insula/Superior temporal gyrus/Postcentral gyrus	R	456	25.74	54	-21	24	<.001
Postcentral gyrus	L	177	23.30	-42	-18	39	<.001

Supplementary Table 2 Simple effects tests comparing functional connectivity between BLA versus CMA in each group.

	Region	diagnosis	BLA	CMA	P value
Left amygdala seed	Left insula	OCD	0.174 (0.019)	0.222 (0.018)	.001
		HC	0.256 (0.019)	0.199 (0.018)	<.001
	Right insula	OCD	0.166 (0.022)	0.223 (0.020)	<.001
		HC	0.212 (0.022)	0.178 (0.020)	.025
Right amygdala seed	SMA/MCC	OCD	0.259 (0.019)	0.314 (0.017)	<.001
		HC	0.305 (0.019)	0.264 (0.017)	.002
	Left STG	OCD	0.301 (0.018)	0.334 (0.018)	.011
		HC	0.319 (0.018)	0.262 (0.018)	<.001
	Right Insula/STG/PCG	OCD	0.271 (0.017)	0.306 (0.017)	.003
		HC	0.302 (0.017)	0.248 (0.017)	<.001
	Left PCG	OCD	0.222 (0.018)	0.217 (0.018)	.692
		HC	0.236 (0.018)	0.148 (0.018)	<.001

Data are presented with mean (SEM). MCC, midcingulate cortex; PCG, postcentral gyrus; SMA, supplementary motor area; STG, superior temporal gyrus.

Supplementary Table 3 Simple effects tests comparing functional connectivity between OCD versus HC in each subregion.

	Region	Subregion	OCD	HC	P value
Left amygdala seed	Left insula	BLA	0.174 (0.019)	0.256 (0.019)	.004*
		CMA	0.222 (0.018)	0.199 (0.018)	.360
	Right insula	BLA	0.166 (0.022)	0.212 (0.022)	.150
		CMA	0.223 (0.020)	0.178 (0.020)	.124
Right amygdala seed	SMA/MCC	BLA	0.259 (0.019)	0.305 (0.019)	.083
		CMA	0.314 (0.017)	0.264 (0.017)	.044
	Left STG	BLA	0.301 (0.018)	0.319 (0.018)	.487
		CMA	0.334 (0.018)	0.262 (0.018)	.006*
	Right Insula/STG/PCG	BLA	0.271 (0.017)	0.302 (0.017)	.201
		CMA	0.306 (0.017)	0.248 (0.017)	.021
	Left PCG	BLA	0.222 (0.018)	0.236 (0.018)	.569
		CMA	0.217 (0.018)	0.148 (0.018)	.009*

Data are presented with mean (SEM). MCC, midcingulate cortex; PCG, postcentral gyrus; SMA, supplementary motor area; STG, superior temporal gyrus. * indicates significance could survive after false discovery rate correction for multiple comparisons.

Supplementary Table 4 Amygdala volume in OCD and HC.

Region	Amygdala volume, mean (SEM)		F	ηp2	$p_{uncorrected}$	$p_{FDR\text{-}corrected}$
	OCD	HC				
Left lateral nucleus	614.6 (6.4)	634.4 (6.5)	4.744	0.026	.031	.062
Left basal nucleus	425.4 (4.7)	432.2 (4.7)	1.048	0.006	.307	.395
Left accessory basal nucleus	241.9 (3.2)	254.8 (3.3)	7.826	0.042	.006	.018
Left anterior amygdaloid area	52.0 (0.7)	53.0 (0.7)	0.892	0.005	.346	.415
Left central nucleus	39.3 (1.0)	42.6 (1.0)	5.489	0.03	.020	.052
Left medial nucleus	21.7 (0.6)	23.7 (0.6)	5.027	0.028	.026	.059
Left cortical nucleus	22.8 (0.5)	25.2 (0.5)	12.662	0.067	<.001	.002
Left corticoamygdaloid transition	182.1 (2.0)	183.7 (2.0)	0.341	0.002	.560	.560
Left paralaminar nucleus	52.6 (0.6)	51.7 (0.6)	1.108	0.006	.294	.395
Left whole amygdala	1652.4 (16.4)	1701.4 (16.6)	4.402	0.024	.037	-
Right lateral nucleus	631.1 (7.3)	648.5 (7.4)	2.755	0.015	.099	.162
Right basal nucleus	445.3 (4.7)	454.7 (4.8)	1.945	0.011	.165	.248
Right accessory basal nucleus	258.0 (3.0)	277.1 (3.0)	20.477	0.104	<.001	<.001
Right anterior amygdaloid area	57.3 (0.8)	58.3 (0.8)	0.727	0.004	.395	.418
Right central nucleus	42.9 (0.8)	47.0 (0.8)	13.842	0.073	<.001	.001
Right medial nucleus	23.6 (0.6)	27.0 (0.6)	17.726	0.091	<.001	<.001
Right cortical nucleus	25.2 (0.4)	28.6 (0.4)	38.725	0.18	<.001	<.001
Right corticoamygdaloid transition	187.9 (2.0)	193.6 (2.0)	3.882	0.021	.050	.091
Right paralaminar nucleus	53.9 (0.7)	53.1 (0.7)	0.776	0.004	.379	.418
Right whole amygdala	1725.2 (17.3)	1787.8 (17.5)	6.42	0.035	.012	-
