# Supplementary material for: Disorganized functional architecture of amygdala subregional networks in obsessive-compulsive disorder
Source: Commun Biol. 2022 Nov 4;5:1184. doi: 10.1038/s42003-022-04115-z (PMC9636402; doi:10.1038/s42003-022-04115-z)
Supplement: Supplementary file 3 — Description of Additional Supplementary Data [file 42003_2022_4115_MOESM3_ESM.pdf]

Description of Additional Supplementary Files

File name: Supplementary Data 1-2

Description: The source data behind the graphs in the paper
