# Supplementary material for: Disorganized functional architecture of amygdala subregional networks in obsessive-compulsive disorder
Source: Commun Biol. 2022 Nov 4;5:1184. doi: 10.1038/s42003-022-04115-z (PMC9636402; doi:10.1038/s42003-022-04115-z)
Supplement: Supplementary file 5 — Reporting Summary [file 42003_2022_4115_MOESM5_ESM.pdf]

nature portfolio

Corresponding author(s): Xiaoqi Huang

Last updated by author(s): Oct 4, 2022

Reporting Summary

Nature Portfolio wishes to improve the reproducibility of the work that we publish. This form provides structure for consistency and transparency in reporting. For further information on Nature Portfolio policies, see our Editorial Policies and the Editorial Policy Checklist.

Statistics

For all statistical analyses, confirm that the following items are present in the figure legend, table legend, main text, or Methods section.

n/a	Confirmed	
☐	☒	The exact sample size (*n*) for each experimental group/condition, given as a discrete number and unit of measurement
☐	☒	A statement on whether measurements were taken from distinct samples or whether the same sample was measured repeatedly
☐	☒	The statistical test(s) used AND whether they are one- or two-sided<br>*Only common tests should be described solely by name; describe more complex techniques in the Methods section.*
☐	☒	A description of all covariates tested
☐	☒	A description of any assumptions or corrections, such as tests of normality and adjustment for multiple comparisons
☐	☒	A full description of the statistical parameters including central tendency (e.g. means) or other basic estimates (e.g. regression coefficient) AND variation (e.g. standard deviation) or associated estimates of uncertainty (e.g. confidence intervals)
☐	☒	For null hypothesis testing, the test statistic (e.g. *F*, *t*, *r*) with confidence intervals, effect sizes, degrees of freedom and *P* value noted<br>*Give P values as exact values whenever suitable.*
☒	☐	For Bayesian analysis, information on the choice of priors and Markov chain Monte Carlo settings
☒	☐	For hierarchical and complex designs, identification of the appropriate level for tests and full reporting of outcomes
☐	☒	Estimates of effect sizes (e.g. Cohen's *d*, Pearson's *r*), indicating how they were calculated

Our web collection on statistics for biologists contains articles on many of the points above.

Software and code

Policy information about availability of computer code

Data collection	3-Tesla GE Signa EXCITE magnetic resonance imaging (MRI) scanner for imaging data.
Data analysis	DPABI, REST, SPM12, CBPtools, FreeSurfer6.0, SPSS24.0

For manuscripts utilizing custom algorithms or software that are central to the research but not yet described in published literature, software must be made available to editors and reviewers. We strongly encourage code deposition in a community repository (e.g. GitHub). See the Nature Portfolio guidelines for submitting code & software for further information.

Data

Policy information about availability of data

All manuscripts must include a data availability statement. This statement should provide the following information, where applicable:

- Accession codes, unique identifiers, or web links for publicly available datasets
- A description of any restrictions on data availability
- For clinical datasets or third party data, please ensure that the statement adheres to our policy

The data that support the findings of this study are available from the corresponding author upon reasonable request.

March 2021

Human research participants

Policy information about studies involving human research participants and Sex and Gender in Research.

Reporting on sex and gender	We used the term sex to indicate biological attribute and sex was determined based on self-reporting. Sex was considered in the group comparison analyses as a covariate.
Population characteristics	There was no significant difference between OCD patients and HC regarding age or sex (P>0.05). All OCD patients had experienced a washout period of at least four weeks before the image acquisition. For the 92 patients with OCD, the total Y-BOCS score was 21.39 ± 5.51, corresponding to moderate and severe OCD symptoms, with obsessive and compulsive scores of 13.01 ± 5.09 and 8.38 ± 5.33, respectively.
Recruitment	OCD patients were recruited from the Mental Health Center, West China Hospital of Sichuan University with diagnosis determined by consensus between two experienced psychiatrists using the Structured Clinical Interview for DSM-IV Axis I Disorders (SCID). HC were recruited from the local area through advertising posters and screened with the SCID (non-patient edition) to confirm the absence of Axis I psychiatric disorders.
Ethics oversight	The Research Ethics Committee of West China Hospital, Sichuan University approved the current study.

Note that full information on the approval of the study protocol must also be provided in the manuscript.

Field-specific reporting

Please select the one below that is the best fit for your research. If you are not sure, read the appropriate sections before making your selection.

☒ Life sciences ☐ Behavioural & social sciences ☐ Ecological, evolutionary & environmental sciences

For a reference copy of the document with all sections, see nature.com/documents/nr-reporting-summary-flat.pdf

Life sciences study design

All studies must disclose on these points even when the disclosure is negative.

Sample size	182
Data exclusions	Participants (OCD patients: 1 out of 93; HC: 3 out of 93) were excluded due to excessive head motion during resting-state MRI data acquisition.
Replication	The results mostly remained consistent when smooth with a 6 mm FWHM Gaussian kernel, no smooth were applied to the imaging data and using an aggressive head motion control strategy (scrubbing).
Randomization	MRI data from 92 OCD patients and 90 healthy controls.
Blinding	Not applicable.

Reporting for specific materials, systems and methods

We require information from authors about some types of materials, experimental systems and methods used in many studies. Here, indicate whether each material, system or method listed is relevant to your study. If you are not sure if a list item applies to your research, read the appropriate section before selecting a response.

Materials & experimental systems

n/a	Involved in the study	
☒	☐	Antibodies
☒	☐	Eukaryotic cell lines
☒	☐	Palaeontology and archaeology
☒	☐	Animals and other organisms
☒	☐	Clinical data
☒	☐	Dual use research of concern

Methods

n/a	Involved in the study	
☒	☐	ChIP-seq
☒	☐	Flow cytometry
☐	☒	MRI-based neuroimaging

Magnetic resonance imaging

Experimental design

Design type	Resting state fMRI and structural MRI.
Design specifications	6min40s of resting-state fMRI sacn.
Behavioral performance measures	No behavioral performance measures.

Acquisition

Imaging type(s)	Functional and structural.
Field strength	3T
Sequence & imaging parameters	Gradient-echo EPI: repetition time (TR) = 2000 ms, echo time (TE) = 30 ms, flip angle = 90°, 30 axial slices with an in-plane voxel resolution of 3.75 × 3.75 mm, 5 mm slice thickness with no slice gap, and filed of view (FOV) = 240 × 240 mm2. Spoiled gradient recall sequence: TR = 8.5 ms, TE = 3.4 ms, flip angle = 12°, 156 contiguous coronal slices of 1.0 mm thickness, and FOV = 240 × 240 mm2 with an acquisition matrix of 256 × 256, which yielded an actual voxel size of 0.93 × 0.93 × 1 mm3.
Area of acquisition	Whole brain.

Diffusion MRI ☐ Used ☒ Not used

Preprocessing

Preprocessing software	DPABI (http://rfmri.org/DPABI).
Normalization	Individual T1-weighted images were co-registered to the mean functional image using a 6 degree-of-freedom linear transformation and then the images were spatially normalized to the standard Montreal Neurological Institute (MNI) template.
Normalization template	MNI template.
Noise and artifact removal	We utilized the Friston 24-parameter model to regress out head motion confounding effects. Furthermore, several sources of nuisance signals (WM signal and CSF signal) were regressed out to reduce the effects of nonneuronal BOLD fluctuations.
Volume censoring	DPABI. We excluded participants with (1) > 1.5 mm of spatial movement (2) > 1.5 degree of rotation in any direction, and (3) mean framewise displacement (FD) > 0.2 mm. In validation analysis, scrubbing (removing time points with FD>0.2 mm) was utilized to verify results when using an aggressive head motion control strategy.

Statistical modeling & inference

Model type and settings	Mixed-effects.
Effect(s) tested	Diagnosis-by-subregion flexible factorial analyses of variance were conducted in left and right amygdala FC separately, with diagnosis(OCD vs. HC) as a between-group factor and subregion as a within-subject factor.

Specify type of analysis: ☒ Whole brain ☐ ROI-based ☐ Both

Statistic type for inference (See Eklund et al. 2016)	Voxel-wise.
Correction	p<0.001 at the voxel level and family-wise-error (FWE) corrections for multiple comparisons with an extent threshold of p<0.025 (0.05/2, amygdala seeds from two hemispheres) at the cluster level.

Models & analysis

n/a	Involved in the study
☐	☒ Functional and/or effective connectivity
☒	☐ Graph analysis
☒	☐ Multivariate modeling or predictive analysis

Functional and/or effective connectivity	Pearson correlation.
